# Barriers in adult vitamin D service provision by health care workers: a qualitative study in three ecologies of Ethiopia

**DOI:** 10.1186/s40795-021-00492-6

**Published:** 2021-12-23

**Authors:** Wubegzier Mekonnen, Yeweyenhareg Feleke, Wubetsh Asnake, Yakob Desalegn, Jemal Haidar, Biruk Lambisso, Tewabech Zewdie

**Affiliations:** 1 School of Public Health, College of Health Science, Addis Ababa University, Addis Ababa, Ethiopia; 2 School of Medicine, College of Health Science, Addis Ababa University, Addis Ababa, Ethiopia; 3 IDRC funded project on the cause of death, Ethiopian Public Health Association, Addis Ababa, Ethiopia; 4 Sante Medical College, Addis Ababa, Ethiopia

**Keywords:** Barriers, Health care providers, Adult, Vitamin D deficiency

## Abstract

**Background:**

Adult vitamin D deficiency, which is a public health problem in low-income countries, is correlated with increased mortality. Although health care workers (HCWs) in Ethiopia are educated on important minerals their counseling on the uses of vitamin D and its possible sources was not witnessed since their knowledge and practice is low. The main aim of this study is to explore barriers to good knowledge, positive attitude, and practice of health care providers on adult vitamin D.

**Methods:**

An exploratory qualitative study is done in three ecologies covering highland, midland, and lowland. The size of 27 participants was fixed by saturation of ideas. In-depth interviews were conducted among leaders and HCWs of different professional groups in health centers and hospitals. Moderators were Ph.D. holders. An interview guide was developed after reviewing research that was translated into the local language. Interviews were audio-taped, transcribed and, translated. Open Code software was used to code and categorize the data. Themes were developed using thematic analysis which is presented using themes and sub-themes.

**Result:**

The main barriers are related to health systems, HCWs’ understandings, and the educational system. Lack of attention by the health systems’ leadership, missing adult vitamin D as a priority in health programs, lack of capacity building scheme and the absence of adult vitamin D management and treatment guidelines are barriers related to the health system*.* On the other hand, health care professionals believed that the prevalence of adult vitamin D deficiency is insignificant and vitamin D deficiency is only an issue related to children. Besides, the absence of studies,the focus of the medical curriculum on child vitamin D and the inadequacy of laboratory investigation are barriers related to the education system and research.

**Conclusion:**

Adult vitamin D deficiency is a neglected public health problem with many barriers related to diagnosis and treatment. Barriers are related to the professionals themselves, their leadership, health facilities, and the education system. The government should give attention to adult vitamin D management and treatment, continuous on the job training, development of guidelines, purchase of laboratory equipment, the inclusion of adult vitamin D in pre-service and, in-service training curriculums.

## Background

Several studies showed that Vitamin D is essential for bone health, extra skeletal tissues, in regulating cell growth and cancer prevention, boosting immune function and infection control and, also in blood pressure regulation and cardiovascular disease. Low vitamin D status has been strongly associated with increased overall mortality [1–3].

Skin synthesis through exposure to ultraviolet B radiation is the vital source of vitamin D. However, even living in the equator does not ensure vitamin D sufficiency [4]. Fatty fish and fish liver oil are among the few natural food sources for vitamin D.

Health care providers should consistently educate their communities on the uses of vitamin D and its possible sources. However, sporadic surveys done in different parts of the World including sub-Saharan Africa indicated that the level of knowledge, attitude, and practice on adult vitamin D deficiency among health care providers is not as expected.

Many health professionals are unable to effectively provide nutrition counseling including vitamin D which may be related to inadequate training, resulting in inadequate knowledge, skills, and low confidence to provide care in nutrition [5, 6]. This problem may arise from the different barriers at different stages of the health system, such as low priority given to nutrition education during medical school, inadequate faculty to provide nutrition training [7, 8].

However, there is limited information with regards to this issue and to the best of the authors’ knowledge no study has been conducted which document the barriers to good knowledge, positive attitude, and practice in the African context which warrants the conduct of such a study in one of the most populous countries with varied agro-ecological zones, Ethiopia.

The main aim of this study is therefore to explore barriers to good knowledge and positive attitude and good practice of health care providers on adult vitamin D in selected ecological zones.

## Methods

This descriptive study employed an exploratory design using purposive sampling to produce qualitative data on barriers to good knowledge, positive attitude, and enhanced practice of health professionals on adult vitamin D deficiency. The study was conducted in three ecological zones covering highland, midland, and lowland (Addis Ababa, Menze-Gera and Qewot Districts) of Ethiopia.

The size of participants for the study was determined based on the saturation of ideas. A total of 27 in-depth face to face interviews were conducted in selected health facilities to understand the perceptions of health care providers on barriers to good knowledge, positive attitude, and good practice on adult vitamin D deficiency. Technical leaders of selected health facilities (medical directors and the like) were recruited as key informants from each selected facility. These participants were identified from different professional groups in health centers and hospitals. Besides, key informants were also recruited from programmers in the health sector including responsible unit head/program officers from the Federal Ministry of Health, regional health bureaus, and Ethiopian Public Health Institute (EPHI). Rapport was built between the research team and study participants before the commencement of the study. Study participants knew most of the research team members as they are closely working with the health sector.

A total of three male Health professionals who hold a Ph.D. in public health having significant experience in conducting qualitative interviews were recruited as data collectors and the principal investigator was their supervisor. Training was provided for the field staff and an intensive supervision was also done.

The questionnaire was developed after reviewing pertinent researches done on the issue. The questionnaire was translated into Amharic. Interviews were semi-structured and took place in a quiet room. They lasted between 30 and 40 min and they were audiotaped. Notes were taken during the interview by an experienced research assistant. The interviews were moderated by the principal investigators of the project. Interviews were started with 29 general questions. Then, probing questions asked, as needed to get a more in-depth understanding of the participants‘ barriers to good knowledge, positive attitude, and practice of health care providers on adult vitamin D. A continuous probing, nondirective and reflective techniques of questions were very important to encourage the participants when they encountered bizarre with the interviews.

No repeat interviews were carried out. Each interview was fully translated and transcribed. Open Code Software was used to code and categorize the qualitative data. Themes were developed from common issues mentioned by the participants. Data were determined to reach theoretical saturation when no new themes or viewpoints were emerging from the interviews. The data was then analyzed using thematic analysis.

Ethical clearance was obtained from Addis Ababa University institutional review board. Support letter was also obtained from the Federal Ministry of Health, the regional health bureaus covering the study ecological zones. Besides, each study participant was asked to give oral consent to participate voluntarily; their privacy was also respected during the interview. In addition to briefing the purpose of the study, respondents were provided with up-to-date knowledge on adult vitamin D deficiency as part of the direct benefit from the particular study.

First, five team members independently reviewed manuscripts from each participant, with the aim to inductively analyze and determine naturally apparent codes. Each member met and discussed apparent themes and agreed on the coding categories that were incorporated into the final coding framework. The remaining transcripts were then analyzed by two members of the study team. Once completed, the team members convened to review overall themes and confirm the findings. Interview data among the different groups that similarly discussed barriers were consolidated when reporting the study findings. Preliminary long-form results were shared four times with the entire team, which provided additional input on interpretation of data to finalize the results below.

The trustworthiness of the results was ensured by careful selection of informants with assistance of recruiters, selection and training of assistant moderator, establishing good rapport with study participants and the use of different data sources and different methods (triangulation). Triangulation of methods was done by collecting data from health professionals working in different levels of health program leadership with particular focus on micro-nutrient program management and leadership; and conducting interviews among those who are involved in awareness creation, diagnosis, management and treatment of adult vitamin D deficiency in different capacities at various levels of health facilities. Besides document review was done to understand available guidelines and procedures on vitamin D diagnosis, management and treatment. Brief explanation on the study objective was given for each study participant to let them discuss freely and participate smoothly. To ensure transferability of data the context in which the research was done, coverage ecological zones (high, midland and lowland), residence types (urban/rural areas) that are inhabited by people with different dressing styles and dietary preferences; and the vitamin D related policy and program environment were thoroughly described.

Transcription of audio information using the local language and translation into English language was commenced concurrently the same day with data collection. Field notes were incorporated with transcripts and familiarization with the data was done. The data collection guides designed based on literatures and adapted to the objectives of the study. The guides-were evaluated carefully and cleared from leading questions. The manuscript was written following the modified checklist of consolidated criteria for reporting qualitative research (COREQ).

## Results

The findings of the study are presented using the main themes and sub-themes against the objective of the study. The main themes include barriers related to the health system, barriers related to health care professionals, and barriers related to the educational system and research which are presented in Table 2.

### Demographic characteristics of study participants

In the study, 27 key informants who had direct relationships and responsibilities with the subject matter were involved. Participants are drawn from the Federal Ministry of Health (FMOH), Zonal health bureau, district health offices, hospitals, and health centers. Their detailed demographic characteristics are provided in Table 1 below.

**Table 1:** Demographic Characteristics of study participants

Variable	Value	Number of participants
Study Region	Menz-Gera	8
	Qewot	7
	Addis Ababa	12
Sex	Male	24
	Female	3
Service year with the current position	< 5	7
	5 to 10	8
	> 10	12
Profession	Nurse	9
	Health officer	8
	Medical doctor	7
	MPH*	3
Place of work	FMOH	1
	Hospital	7
	Health center	14
	Zonal and district health office	4
	EPHI	1

*MPH = Masters of Public Health

The key findings of the study are presented hereunder by thematic and sub-thematic areas which are revealed in Table 2.

**Table 2:** Table showing themes and associated quotes, barriers to adult vitamin D deficiency diagnosis, management and treatment in three ecologies of Ethiopia

Thematic barriers related to:	Sub-thematic barriers
the health system	1. Lack of attention to adult vitamin D deficiency at different levels 2. Adult vitamin D Deficiency is not included in routine health program or report 3. No guideline or manual prepared by health bureaus for adult vitamin D deficiency management 4. No capacity building scheme
health professionals	1. Health professionals believe that the prevalence of adult vitamin D is very low 2. Vitamin D deficiency is perceived to be a common problem among children not in adults
the education system and research	1. Compromised pre-service training 2. Health curriculum focused only on child vitamin D deficiency 3. No adequate research to show the magnitude of adult vitamin D deficiency 4. No adequate lab investigation

### Barriers related to the health system

The Federal Ministry of Health is responsible to formulate health policy, set standards to deliver health care in Ethiopia, and provide strategic direction to health delivery services. Failure in commitment and inadequate understanding of health problem by higher officials will result in poor service provision at lower-level health facilities.

#### Lack of attention to adult vitamin D deficiency at federal, regional, zonal, and district level

Unlike other micronutrients, adequate attention is not given to vitamin D deficiency especially adult vitamin D is ignored as a program. Adequate attention is not given to adult vitamin D in federal, zonal and district program leadership levels leave alone at the lower level of health facilities. In line with this, the respondent from the ministry of health mentioned that *“In adults, I have not seen any commitment and efforts to do interventions to alleviate problems related to vitamin D deficiency.” (k27).*

The city administration and district health offices did not also incorporate vitamin D related activities under their programs and are not given any support or supportive supervision related to the prevention and treatment of adult vitamin D deficiency management. The respondent from Addis Ababa health bureau stated that *“The problem of adult vitamin D did not get the required attention from the government. In fact, I do not know what health facilities are doing. But in my department, we are not doing anything related to adult vitamin D deficiency.” (k26).*

Another respondent from Menze-Gera district also commented that awareness creation was not done by higher officials in the health sector and the area is not well studied. The respondent said “*No awareness was created from top management to health care providers. It is not known whether the problem of adult vitamin D deficiency exists. It has not even been studied.” (k01).*

#### Adult vitamin D is not included in routine program or report

Unlike other micronutrients’ deficiencies, vitamin D deficiency especially among the adult population is a neglected area. Study participants believe that the reason for the low levels of knowledge, attitude, and practice of adult vitamin D deficiency is related to the low attention and support given by zonal health departments and district health offices. One of the respondents from the Arada health center in Addis Ababa mentioned that *“When senior officials come to health centers for supportive follow up supervision, they have checklists for Iron and vitamin A deficiency; they will do the same for vitamin D deficiency if they give it enough attention.” (k20).*

There are sixteen packages that are included in the health extension program (a widely advocated community health program in Ethiopia); unfortunately, adult vitamin D is not included in the list. The respondent from the *Feresmeda* health center in Addis Ababa mentioned that adult vitamin D is not included in the outreach programs of primary health care workers. This is what she has to say in her own words *“This health center is famous in outreach activities. Since the inception of primary health care program by the government, vitamin D is not included. We use different formats to document health problems and conditions in different tiers of the health care system; vitamin D is not among the list.” (22).*

Vitamin D deficiency is not also included under monthly reportable health problems and conditions by health centers and hospitals in their monthly Health Management Information System (HMIS) reporting format. The respondent from Menze-Gera also stated that *“We do not include adult Vitamin D in our plan and have not been included in our report, or reported it on our monthly HMIS reporting.” (k02).*

#### No guidelines or manual prepared by health system for adult vitamin D management

Lack of guidelines or manuals prepared on adult vitamin D deficiency management and treatment is also mentioned as one of the reasons for the low level of knowledge, attitude, and practice of health care professionals on the diagnosis, management, and treatment of adult patients with vitamin D deficiency. The respondent from the federal ministry of health witnessed that there is no separate guideline for vitamin D management and treatment*.* According to the key informant *“There is no clear guideline about Vitamin D deficiency diagnosis, treatment and management.” (k27).* He also added that, even in the guideline for micronutrients, enough attention is not given to adult vitamin D deficiency and only two statements described vitamin D deficiency in the micronutrient guideline. Here is what he said *“Yes, there is a mention of vitamin D but there are only two lines within the micronutrient guideline.” (k27).*

A respondent from the Ethiopian Public Health Institute, which is the research wing of the ministry of health complemented and mentioned low attention given by government as the reason for not having a separate guideline for vitamin D diagnosis, management, and treatment. He said *“…we did not prepare adult vitamin D deficiency guideline and there is no effort to have it soon. So, we do not give the required attention and address the problem.” (k25).*

Moreover, study participants who are front line health care workers in different health facilities confirmed the views of program leaders. A respondent from Feresmeda health center in Addis Ababa replied the following when asked if there is any manual or guideline for adult vitamin D deficiency *“To tell you the truth, there is none.” (k22).* Another respondent from Menze-Gera also stated *“There is also a problem with the diagnostic system as we do not have guidelines for the investigation of the same.” (k03)* The same respondent also added that he does not know whether there is a separate guideline for vitamin D. *“There is no dedicated guideline for vitamin D.” (k03).*

#### No capacity building scheme

On the job training is one way of building the capacity of health care professionals in different health-related programs. However, participants of this study reiterated that no in-service training or capacity building on adult vitamin D deficiency diagnosis, management, and treatment was given to them in recent years. The participant from Hayat public health center in Addis Ababa stated that *“the knowledge gap regarding adult vitamin D deficiency is related to the absence of on job training in the area”.* The respondent when asked about the reason that he never experience any case of adult vitamin D deficiency replied that *“Maybe it’s because of lack of capacity to diagnose adult vitamin D deficiency due to lack of training and awareness.” (k19).*

Another key informant from Menze-Gera health center also said that he did not receive in-service training on adult vitamin D deficiency diagnosis, management and treatment since his employment. He described the context as*“… I didn’t receive any training on adult vitamin D diagnosis, management and treatment. I have the capacity to identify vitamin D deficiency among children. But on adults I am less likely to do that…” (k06).*

Key informants working in health program do not have a different view. A respondent from North Showa health department reiterated the issue as follows *“By chance, I have taken few courses on vitamin D while I was in college, however I do not have a chance to receive in-service training on vitamin D in general, and adult vitamin D deficiency diagnosis, management and treatment in particular thereafter.” (k07).*

The issue is not also different for health care providers working in the capital, Addis Ababa. A respondent from Saris health center, in Addis Ababa, said that *“I recommend that skill and capacity of health professionals on adult vitamin D deficiency diagnosis, management and treatment should develop through training as there is none who received such a training in our facility.” (k23).*

### Barriers related to health professionals

Health care professionals play a central and critical role in improving access and quality of health care in a community by promoting good health, educating people on disease prevention, and working on health care provision services to individuals and families. So, any knowledge gap in this vital group may result in poor utilization of health care services that leads to poor quality of life in the wider community at large.

#### Health professionals believe that the prevalence of adult vitamin D deficiency is very low

Study participants believe that the prevalence of vitamin D in the general population and its deficiency among adults is very minimal in Ethiopia as the country is usually known for its thirteen months (including the leap year) of sunshine. Thus, adult vitamin D deficiency does not require special attention, unlike other micronutrient deficiencies.

One of the respondents from Qewot district, which is a lowland area, working at a health center when asked about the prevalence of vitamin D in adult population replied that *“Till now there is no information on adult vitamin D deficiency in our district. No research was done and no lab confirmation on adult vitamin D deficiency to the best of my knowledge.” (k09).*

Another respondent also added that *“The vitamin D deficiency problem is not a significant public health problem in our area, in my entire work experience I have seen only two cases of vitamin D deficiency.” (K10).*

On the other hand, others argued that the low prevalence of adult vitamin D reported by Ethiopian health facilities might be related to the knowledge gap of health care professionals. A respondent from the Feresmeda health center in Addis Ababa mentioned that “*They may not come to us since we may not give them proper treatment or health professionals may not be able to correctly diagnose the deficiency. Until now, we haven’t seen any single case” (k22).*

Not only the wider community but also health professionals do not have an adequate awareness on adult vitamin D deficiency and we are not practicing anything related to it. A health care provider from the Saris health center in Addis Ababa reiterated the issue *“To tell you the truth, there is no work related to adult vitamin D in our facility at all. There is no capacity building given to staff. We do not have the awareness about the problem from the beginning.” (k23).*

#### Vitamin D deficiency is perceived to be a common problem among children not in adults

Most health care providers mentioned that vitamin D deficiency is a major public health problem among children. They do not consider it as a problem among adults. One of the health care providers from *Feresmeda* health center mentioned that: *“Most of the health care workers don’t have an adequate awareness, they consider vitamin D deficiency as a problem for children, but not for adults.” (k22).*

The same view is shared among professionals working in health program leadership. The respondent from the federal ministry of health mentioned that some health professionals do not have a clue on adult vitamin D deficiency. *“Health care providers not only have problems to diagnose and treat adult vitamin D deficiency but they did not also know adult vitamin D deficiency as a public health program.” (k27).*

The respondent from the research wing of the Ministry of Health, EPHI, has the same view. He said *“Attention is given to children vitamin D deficiency but not to the deficiency among adults. Health care providers in each health facility did not give adult vitamin D deficiency the required attention.’ (k25).*

The same respondent also added that because adults spend most of their time in outdoor activities, they have enough access to sunlight exposure, so they are at less risk for vitamin D deficiency. Here is what he said in his own words “*Most of the adults unlike children stay outside their home and have the chance of getting sunlight exposure. As a result, there is no report on adult vitamin D deficiency and the lack of evidence makes people not to consider the problem among adults” (k25).*

The Addis Ababa city administration health bureau staff has a similar understanding on adult vitamin D deficiency which is reiterated as follows. *“I do not know that adult vitamin D deficiency is a public health problem in our area.” (k26).*

### Barriers related to the education system and research

#### Compromised pre-service training

Most of the respondents believe that their knowledge gap related to adult vitamin D deficiency is attributed to the failure of the curriculum to incorporate a session on adult vitamin D in micro-nutrient trainings.

One of the participants from Arada health center in Addis Ababa mentioned the following with regards to pre-service training *“No. there was no session on adult vitamin D deficiency diagnosis, management and treatment in our college level training. That is why we fail to handle issues related to adult vitamin D deficiency” (k20).*

It is complemented by the view of another study participant recruited from a health center in Menze-Gera district in Amhara region *“While we were in college, we didn’t learn how to measure vitamin D status among adults and know its deficiency.” (k05).*

#### Health curriculum focused only on child vitamin D deficiency

Study participants indicated that even the sessions on low credit hour course on micronutrients including vitamin D focused on the deficiency of vitamin D on children without mentioning such deficiency among adults. The key informant from EPHI mentioned that “*All training programs related to vitamin D deficiency is focusing on child vitamin D deficiency. The curriculum content did not take in to account the adult vitamin D deficiency.” (k25).*

However, the contents of sessions on vitamin D training curriculum varies by the training level of health care providers. A study participant from Menelik II memorial hospital indicated that he has received trainings on vitamin D although the focus was more on child vitamin D deficiency as follows *“We learned about vitamin D deficiency and it is well addressed in the curriculum. But the details for adult vitamin D deficiency are not like the one given for child vitamin D deficiency. The training indicated that vitamin D deficiency exists both in children and adults, though details on diagnostic procedures, management and treatment particularly for adults was not given.” (k21).*

#### No adequate research is done to show the magnitude of adult vitamin D deficiency

No supporting research is done to show the magnitude of adult vitamin D deficiency in Ethiopia. The study participant from EPHI, the research wing of the ministry of health, mentioned that because there is no research done in the area it is difficult to inform programs and policies. Here is what he said “*Like other vitamins, our institution has the capacity and all the required facility, equipment and supplies to conduct research on vitamin D deficiency and other researches on other minerals. But there is no detailed research done on vitamin D deficiency. We did not know the magnitude and its distribution.” (k25).*

An expert from Menelik II memorial hospital supported the view of the previous respondent as follows *“To conclude whether it is a public health problem of the community, there should be detailed research done in the community. We are not doing screening activities. We do not have any kind of data on adult vitamin D deficiency condition, either.” (k21).*

#### No adequate laboratory investigation

The frequent problem mentioned by study participants that hinder them from identifying and diagnosing vitamin D among the adult population is related to absence of laboratory equipment to diagnose vitamin D status in lower-level health facilities.

A respondent from Menze-Gera district, the coldest high land district selected for the particular study mentioned that *“We have no adequate resource to purchase equipment to diagnose adult vitamin D deficiency; there is no diagnostic equipment to conduct laboratory testing.” (k04).*

Resource shortage was also mentioned by a hospital staff in Addis Ababa. A study participant from Yekatit 12 memorial hospital in Addis Ababa also said *“… For example, we do not have electrolyte test in this facility since we don’t have the machine to test it.” (k24).*

## Discussion

In this study, we assessed barriers to good knowledge, positive attitude, and enhanced practice of health care providers on adult vitamin D Deficiency, management and treatment in highland, lowland, and midland ecologies in Ethiopia.

Lack of attention by higher officials in the health sector, lack of consideration of adult vitamin D in routine health programs, and absence of separate guidelines for the diagnosis, management and, treatment of adult vitamin D deficiency were barriers related to the health care system in Ethiopia. Whereas, health professionals believe that there is low prevalence of vitamin D among adults and it is common only in children, which are barriers related to the health care providers in Ethiopia. Moreover, limited research is done on adult vitamin D deficiency. Besides, the focus of medical curriculum is only on child vitamin D deficiency; there is inadequate pre-service training and inadequate laboratory investigation is documented as barriers related to the educational system in the country.

This study witnessed that health educational curricula do not sufficiently address adult vitamin D to give health science students detailed knowledge in adult vitamin D deficiency. Similar research in Ghana also shows that around 66% of doctors who participated in the study said they had inadequate nutrition education during medical school, and more than 70% of the participants were either unsatisfied or undecided regarding the quality and quantity of their nutrition educational experiences during medical school [9] which is also similar with other study done in USA and Australia [10, 11].

In addition to the pre-service courses, on the job training is believed to be a major anchor in developing knowledge and enhancing skill in clinical practice. But this study found that on job training and capacity-building mechanisms on adult vitamin D deficiency are non-existent that might have a significant role in the knowledge gap, attitude, and low practice of health care professionals on the diagnosis, management, and treatment of adult vitamin D deficiency. Similar studies that were done in other countries also confirm this finding. A study done in Ghana shows that the majority of health care workers improve their competence related to nutrition through reading and self-directed learning. Only a very minimal number (12.1%) of the participant mentioned conferences/workshops as a means of capacity building for knowledge related to nutrition [9].

Another important finding of this study was that even the higher official from the federal ministry of health admits the absence of guidelines related to adult vitamin D deficiency. It is known that guidelines are systematically developed statements that help health care professionals to easily diagnose and treat patients and make decision making better and easy. But we do not have such a tool for adult vitamin D deficiency in Ethiopia. Even when you have such guidelines there should be tailored training on it as a study done in Canada confirm low utilization of guidelines by health care professionals [12] and another study done in Ghana also confirmed that only 42% of health care professional use guidelines to diagnose and treat adult vitamin D deficiency [9]. This finding gives an insight that the federal ministry of health can and should have done a lot in the preparation and distribution of guidelines on adult vitamin D deficiency.

According to the recent study done among the adult population in southern Ethiopia in 2013, only 15.8% of the participants had 25(OH) D levels above 50 nmol/L, which shows a high prevalence of adult vitamin D deficiency in the community [13]. However, this study found that one of the barriers for low knowledge, attitude, and poor practice of adult vitamin D deficiency was related to inappropriate and incorrect assumption given by the health care professionals that vitamin D is a common condition among children than adults. This false assumption may come from the absence of studies that show the actual magnitude, inadequate pre-service training, low attention given by the health system leaders at federal, regional as well as zonal, and district levels, and limited availability of laboratory equipment and supplies in health facilities.

Generally, since this is the first study done in Ethiopia, it is believed that it will give baseline information on barriers for good knowledge, positive attitude, and enhanced practice of health professionals on adult vitamin D deficiency. And since this is a qualitative study, it gives clear and explanatory details in the area and also will be used as a reference for future studies.

Inclusion of respondents from different professional hierarchy (from the health center to ministry of health) and taking samples from different ecological zones (low land, midland and high land) and residence types (urban and rural) can be cited as the strength of the study. However, the study may benefit if it is done in several regions to increase its transferability which can be cited as a limitation of the study.

## Conclusion

In conclusion, adult vitamin D deficiency is a totally neglected area in the health sector. Low attention is given to it by the health professionals working in program leadership and health care practice at national, regional, and district levels. Moreover, medical curriculums do not adequately include adult vitamin D, there is inadequate laboratory investigations in health facilities. Besides, there is a knowledge gap since health professionals think that adult vitamin D is common in children and not in adults in addition to thinking that adult vitamin D deficiency is a rare problem. Very limited research is done in the area. There are no guidelines on adult vitamin D deficiency diagnosis, management, and treatment, and subsequent on the job training. Besides, there is no organization supporting the adult vitamin D program.

Appropriate attention needs to be given by the health system leadership which has to be cascaded to lower levels. Besides, frequent in-service training should be given in addition to revisiting the medical curriculum to incorporate adult vitamin D deficiency. The laboratories in health facilities should be equipped with diagnostic kits and there should be adequate training of staff to improve their skills. Intervention studies have to be done to get more evidence on the issue under caption. Finally, like other programs, partners should participate in designing appropriate programs related to adult vitamin D deficiency.

### Abbreviations/acronyms

HCWs Health Care Workers

Ph.D.Philosophy of Doctorate Degree.

EPHI Ethiopian Public Health Institute.

EPHA Ethiopian Public Health Association.

FMOH Federal Ministry of Health.

MPH Masters of Public Health.

K01Key Informant 1.

HMIS Health Management Information System.

USAUnited States of America.

OH Hydrogen Oxide.

Nmo/L Nanomoles per litre.

IRB Institutional review Board.

SPH School of Public Health.

REC Research Ethics Committee.

CHS College of Health Sciences.

AAU Addis Ababa University.
